# Intimal sarcomas and undifferentiated cardiac sarcomas carry mutually exclusive *MDM2*, *MDM4*, and *CDK6* amplifications and share a common DNA methylation signature

**DOI:** 10.1038/s41379-021-00874-y

**Published:** 2021-07-26

**Authors:** Christian Koelsche, Jamal K. Benhamida, Felix K. F. Kommoss, Damian Stichel, David T. W. Jones, Stefan M. Pfister, Christoph E. Heilig, Stefan Fröhling, Albrecht Stenzinger, Rolf Buslei, Thomas Mentzel, Daniel Baumhoer, Marc Ladanyi, Cristina R. Antonescu, Uta Flucke, Joost van Gorp, Beata Bode-Lesniewska, Andreas von Deimling, Gunhild Mechtersheimer

**Affiliations:** 1 Department of General Pathology, Institute of Pathology, University Hospital Heidelberg, Heidelberg, Germany; 2 Department of Pathology, Memorial Sloan Kettering Cancer Center, New York, USA; 3 Department of Neuropathology, Institute of Pathology, University Hospital Heidelberg, Heidelberg, Germany; 4 Clinical Cooperation Unit Neuropathology, German Cancer Consortium (DKTK), German Cancer Research Center (DKFZ), Heidelberg, Germany; 5 Hopp Children’s Cancer Center Heidelberg (KiTZ), Heidelberg, Germany; 6 Paediatric Glioma Research Group, German Cancer Consortium (DKTK), German Cancer Research Center (DKFZ), Heidelberg, Germany; 7 Division of Pediatric Neurooncology, German Cancer Consortium (DKTK), German Cancer Research Center (DKFZ), Heidelberg, Germany; 8 Department of Pediatric Oncology, Hematology and Immunology, Heidelberg University Hospital, Heidelberg, Germany; 9 Division of Translational Medical Oncology, National Center for Tumor Diseases (NCT) Heidelberg and German Cancer Research Center (DKFZ), German Cancer Consortium (DKTK), Heidelberg, Germany; 10 Institute of Pathology, Sozialstiftung Bamberg, Bamberg, Germany; 11 Dermatopathology Bodensee, Friedrichshafen, Germany; 12 Bone Tumor Reference Center at the Institute of Medical Genetics and Pathology, University Hospital and University of Basel, Basel, Switzerland; 13 Department of Pathology, Radboud University Hospital, Nijmegen, the Netherlands; 14 Department of Pathology, St Antonius Hospital Nieuwegein, Nieuwegein, the Netherlands; 15 Department of Pathology, University Hospital, Zurich, Switzerland

**Keywords:** Diagnostic markers, Cancer

## Abstract

Undifferentiated mesenchymal tumors arising from the inner lining (intima) of large arteries are classified as intimal sarcomas (ISA) with *MDM2* amplification as their molecular hallmark. Interestingly, undifferentiated pleomorphic sarcomas (UPS) of the heart have recently been suggested to represent the cardiac analog of ISA due to morphological overlap and high prevalence of *MDM2* amplifications in both neoplasms. However, little is known about ISAs and cardiac UPS without *MDM2* amplifications and molecular data supporting their common classification is sparse. Here, we report a series of 35 cases comprising 25 ISAs of the pulmonary artery, one ISA of the renal artery and 9 UPS of the left atrium. Tumors were analyzed utilizing the Illumina Infinium MethylationEPIC BeadChip array, enabling copy number profile generation and unsupervised DNA methylation analysis. DNA methylation patterns were investigated using t-distributed stochastic neighbor embedding (t-SNE) analysis. Histologically, all ISAs and UPS of the left atrium resembled extra-cardiac UPS. All cases exhibited highly complex karyotypes with overlapping patterns between ISA and UPS. 29/35 cases showed mutually exclusive amplifications in the cell-cycle associated oncogenes *MDM2* (25/35), *MDM4* (2/35), and *CDK6* (2/35). We further observed recurrent co-amplifications in *PDGFRA* (21/35), *CDK4* (15/35), *TERT* (11/35), *HDAC9* (9/35), and *CCND1* (4/35). Sporadic co-amplifications occurred in *MYC*, *MYCN,* and *MET* (each 1/35). The tumor suppressor *CDKN2A/B* was frequently deleted (10/35). Interestingly, DNA methylation profiling (t-SNE) revealed an overlap of ISA and cardiac UPS. This “ISA” methylation signature was distinct from potential histologic and molecular mimics. In conclusion, our data reveal *MDM4* and *CDK6* amplifications in ISAs and UPS of the left atrium, lacking *MDM2* amplification. We further report novel co-amplifications of various oncogenes, which may have therapeutic implications. Finally, the genetic and epigenetic concordance of ISAs and UPS of the left atrium further supports a shared pathogenesis and common classification.

## Introduction

Intimal sarcoma (ISA) is an exceedingly rare undifferentiated sarcoma that arises in the pulmonary artery and less frequently in the aorta or its branches [1]. ISAs behave highly aggressive with a mean patients’ survival ranging from 5 to 18 months [1]. Patients with ISA are mostly of middle age at diagnosis and typically present with non-specific symptoms, which sometimes masquerades as thromboembolic disease. Thus, patients are often diagnosed in an advanced disease stage. Furthermore, ISAs are often reported to be resistant to conventional chemotherapy [2].

First recognized by Mandelstamm as pulmonary artery sarcoma from an autopsy in 1923, case reports and small case series have been published for the following decades [3]. The term “intimal” was coined with reference to the attributes that these sarcomas arise from the subendothelial space of arteries, form polypoidal and endoluminal protrusions and spread laterally along the intima of large arteries before they infiltrate beyond the adventitia. However, it has also been noted that sarcomas other than ISA may occasionally exhibit such “intimal” growth pattern [4, 5].

Histologically, ISAs are composed of spindle shaped, pleomorphic or epithelioid cells often resembling soft tissue correlates like undifferentiated pleomorphic sarcoma (UPS), myxofibrosarcoma or epithelioid angiosarcoma. By definition, ISAs lack specific lineage differentiation, although myofibroblastic and rarely osteogenic or chondroid differentiation may occur [5–7].

Molecular studies on pulmonary artery ISAs revealed a high frequency of *MDM2* amplifications, accompanied by co-amplifications of *CDK4* and *PDGFRA* [8–15]. In 2014, Neuville et al. discovered *MDM2* amplifications in a large proportion of UPS of the heart. Interestingly, many of the *MDM2* amplified cardiac UPS presented with histologic features resembling ISA [13]. The authors proposed the concept that these cardiac UPS may represent ISA [16]. However, concerns rose with regards to the non-specific occurrence of *MDM2* amplifications in a broad range of tumor types [10]. Moreover, genome-wide copy number analysis has been performed only in a few pulmonary artery ISAs, whereas ISAs of more uncommon sites such as the aorta have not been analyzed to date (Table 1). It was concluded that reclassifying these cardiac UPS as ISA could be premature, besides that fact that the term “intima” would be inappropriate for these tumors from an anatomic point of view [17, 18]. Thus, the 4^th^ edition of the WHO classification of tumors of the heart differentiates the multiple subtypes of cardiac UPS by histopathology, but mentions ISA as a synonym or alternative designation [19, 20]. Thus, the relationship between arterial and cardiac ISAs remains incompletely understood.

**Table 1:** Studies applying genome-wide copy number variation analysis in intimal sarcomas.

Study	Year	Analyzed cases	Location	Method	Relevant findings
Bode-Lesniewska et al.	2001	8	Pulmonary artery	CGH	*MDM2* amp
Zhao et al.	2002	8	Pulmonary artery	aCGH	*MDM2* amp, *PDGFRA* amp
Sebenik et al.	2005	12/14	Aorta and branches	CGH	Complex karyotyp
Zhang et al.	2007	1	Heart	Karyotyping	*MDM2* amp, *CDK4* amp
Dewaele et al.	2010	8/21	Pulmonary artery (*n* = 5) and heart (*n* = 3)	aCGH	*MDM2* amp, *PDGFRA* amp
Neuville et al.	2014	5/100	Heart	aCGH	*MDM2* amp
Ito et al.	2017	1	Heart	aCGH	*MDM2* amp, *PDGFRA* amp
Roszik et al.	2019	13	Unknown	GENIE database	*MDM2* amp, *TERT* amp

*aCGH* microarray-based comparative genomic hybridization, *amp* amplified.

High density DNA methylation arrays provide a powerful tool for robust molecular tumor classification [21, 22]. DNA methylation profiling in sarcomas has defined subtype-specific sarcoma signatures, even within seemingly morphological homogenous entities that would otherwise evade a definite histologic diagnosis. Likewise, DNA methylation profiling has shown morphological heterogeneous tumors to constitute a single molecular subtype [23–29]. Furthermore, data of these high-density DNA methylation arrays allow genome-wide mapping of copy number variations.

Herein, we comprehensively characterize a cohort of 26 ISAs and 9 UPS of the left atrium by genome-wide copy number analysis and DNA methylation profiling. We sought to further define their molecular alterations and determine whether they share a DNA methylation signature that segregates them from potential histologic mimics.

## Material and methods

### Sample selection

We collected a cohort of 26 ISAs and 9 UPS of the left atrium from different patients, containing 10 previously published cases [8, 9]. Samples were retrieved from the Institute of Pathology of the University Zürich (Switzerland), from the Department of Pathology of the St Antonius Hospital Nieuwegein (the Netherlands), the Institute of Pathology of the University Hospital Heidelberg (Germany), the Department of Pathology of the Memorial Sloan Kettering Cancer Center in New York (United States of America), the Department of Pathology of the Radboud University Medical Center in Nijmegen (the Netherlands), the Dermatopathology Bodensee in Friedrichshafen (Germany) and the Institute of Pathology of the Sozialstiftung Hospital in Bamberg (Germany). Basic clinical information of the investigated cases is provided in Supplementary Table 1.

Diagnoses were established according to the guidelines of the WHO classification for soft tissue and bone tumors (5^th^ edition) and for tumors of the lung, pleura, thymus and heart (4^th^ edition) [1, 19]. Accordingly, none of the study cases showed a definable line of differentiation. The study was performed in concordance with the guidelines set forth by the local ethics committee of the University of Heidelberg and in accordance with the Declaration of Helsinki.

### DNA extraction and quantification

DNA of all tumors was extracted from formalin-fixed paraffin-embedded (FFPE) tissue samples. All tumors included in this study had sufficient tumor material available to prevent extraction of neighboring benign tissue. Areas with highest available tumor content (≥70%) were chosen for extraction of DNA. The Maxwell® 16 FFPE Plus LEV DNA Kit was applied on the automated Maxwell device (Promega, Madison, WI, USA) according to the manufacturer’s instructions. Extracted DNA was quantified using the QuantiFast SYBR Green PCR Kit (Qiagen, Duesseldorf, NW, Germany). A minimum of 100 ng DNA was extracted in every case and provided for subsequent array-based DNA methylation analysis.

### Genome-wide DNA methylation data generation and pre-processing

The total DNA input suitable for DNA methylation profiling ranges from a minimum of 10 ng to 500 ng [30]. In this study, all 35 samples reached a total DNA input of ≥100 ng and therefore were subjected to the Illumina Infinium MethylationEPIC BeadChip array (Illumina, San Diego, USA) analysis at the Genomics and Proteomics Core Facility of the German Cancer Research Center (DKFZ) Heidelberg. To exclude low-quality samples from the cohort, the on-chip quality metrics of all samples were checked and compared to a set of 7,500 pairs of IDAT-files [22]. All 35 samples passed this quality control check. DNA methylation data were normalized by performing background correction and dye bias correction (shifting of negative control probe mean intensity to zero and scaling of normalization control probe mean intensity to 20000, respectively). Probes targeting sex chromosomes, probes containing multiple single nucleotide polymorphisms and those that could not be uniquely mapped were removed. Human reference genome (hg19) was used for the analysis of multi-site mapping. Probes from the EPIC array were excluded if the predecessor Illumina Infinium 450k BeadChip did not cover them, thereby making data generated by both 450k and EPIC feasible for subsequent analyses. In total, 438370 probes were kept for analysis.

### Copy number analysis

Copy number plots were generated on methylation array data using the R package ‘conumee’ after additional baseline correction (https://github.com/dstichel/conumee). Copy number variants were identified by manual inspection as previously described [31]. Thresholds for the identification of amplifications and homozygous deletions were derived from the difference of the baseline. Gains/amplifications usually are above a log2 value of 0.4 and losses/deletions usually are below a log2 value of 0.4. Low tumor cell content or subclonal alterations may reduce the amplitude deviation.

### Fluorescence In Situ Hybridization

Additional FISH assays were performed for assessing gene copy alterations. FISH on interphase nuclei from FFPE 4 μm sections was performed using custom-designed probes of bacterial artificial chromosomes flanking the target genes *CDK6* (7q21.2) and *MDM4* (1q32.1). An amplification was defined as the presence of >10 signals (ratio to control probe >10) or tight clustered signals characteristic of homogeneous staining regions.

### Unsupervised DNA methylation analysis

We used t-distributed stochastic neighbor embedding (t-SNE) analysis, a method enabling dimensionality reduction and visualization of clusters to detect methylation clusters. The algorithm was performed using the 10,000 most variable probes with a perplexity of 10 and 3000 iterations. Methylation groups were tested for stability by varying the number of the most variable probes and perplexities. Methylation data from 78 samples previously published in part comprising non-radiation /–UV induced angiosarcomas of soft tissue or viscera (*n* = 6), inflammatory myofibroblastic tumors (*n* = 7), leiomyosarcomas of (venous) vessels (*n* = 12), conventional high grade osteosarcomas (*n* = 13), low grade osteosarcomas, *MDM2* amplified (*n* = 6), extraskeletal osteosarcomas (*n* = 4), retroperitoneal well-/de-differentiated liposarcomas, *MDM2* amplified (*n* = 13), angiomatoid fibrous histiocytomas (*n* = 9) and extra-cardiac UPS (*n* = 8) were used for comparison [23–29].

## Results

### Study cohort and histopathology

The study included 25 cases with ISAs arising in the pulmonary artery and one case with an ISA of the renal artery. The sex ratio was balanced (male-female ratio, 13:13). Patient’s age at presentation ranged from 30 to 83 years, with a median age of 58 years. Furthermore, the study included 5 female and 4 male patients with UPS of the left atrium, among them one young patient with a metastatic bone lesion in the humerus, which was analyzed here. Their age at presentation ranged from 18 to 67 years, with a median age of 38 years. The patient’s characteristics are summarized in Table 2.

**Table 2:** Patient characteristics and histologic features.

Variables	Artery (*n* = 26)	Heart (*n* = 9)
Sex		
Male	13	4
Female	13	5
Median Age in years (range)	58 (30–83)	38 (18–67)
Histological subtype		
UPS-like	23	9
Malignant IMT-like	1	0
AFH-like	1	0
Myxofibrosarcoma	1	0

**Fig. 1:**
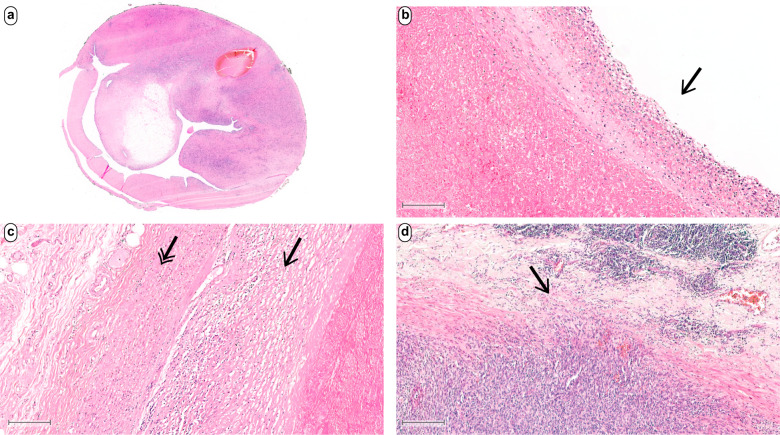
Examples of characteristic architectural patterns in intimal sarcomas. At low magnification this case illustrates the endoluminal growth of a polypoid tumor within an artery (**a**). Intimal sarcomas typically overgrow fibrin layers attached at the vessel wall (**b**). Tumor cells spread lateral within the intimal space (**c**). Some cases show focal infiltration and penetration of the tunica media (**d**). Scale bars equal 200 µm.

**Fig. 2:**
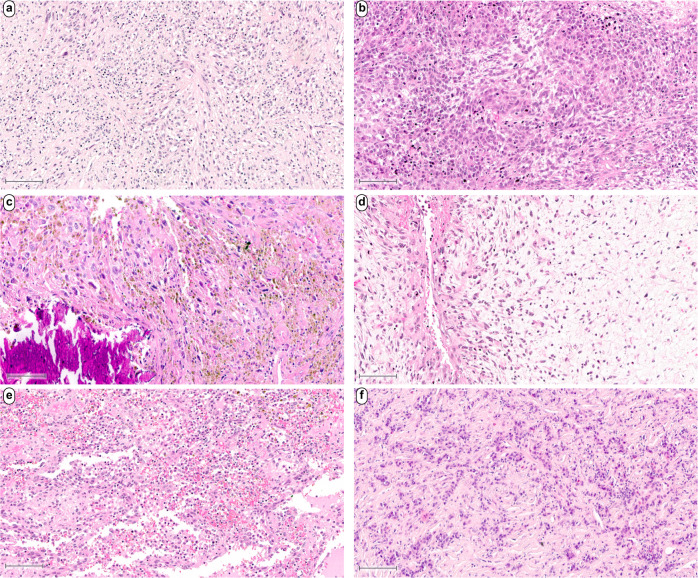
Histologic features in intimal sarcomas. Intimal sarcomas often exhibited a loose storiform growth pattern (**a**). Single cases showed an epithelioid cytomorphology with tumor cells focally forming diffuse sheets and solid areas (**b**). Some cases showed focal stromal sclerosis, hemosiderin deposits and dystrophic calcifications (**c**). Case 141642 exhibited a prominent myxoid stroma with elongated, thin-walled vessels and increased perivascular tumor cell density (**d**). Case 141634 showed variable-sized pseudocystic spaces containing homogeneous eosinophilic material or blood. These spaces are lined by tumor cells. Hemosiderin is present (**e**). Case 129604 showed a prominent component of aggregating plasma cells (**f**). Scale bars equal 100 µm.

Morphologically, all 35 cases, some at least focally, showed ISA features characterized by endoluminal growth, fibrin layering with tumor overgrowth and intimal spread (Fig. 1). Overall, the cellularity was variable within tumors. The predominant tumor architecture was loose and pattern-less, although storiform areas and collagenized stroma were recognizable in some cases. The tumor cells appeared mostly spindle shaped (Fig. 2a) and rarely epithelioid (Fig. 2b). Nuclear pleomorphism was evident in all cases. Some case showed prominent bleeding residues and two cases presented with dystrophic calcifications (Fig. 2c). Three cases exhibited patterns reminiscent of distinct soft tissue sarcoma subtypes other than undifferentiated (pleomorphic) sarcoma. One case (ID 141642) presented with myxoid areas with low cellularity, but tumor cell condensation around vessels, resembling myxofibrosarcoma (Fig. 2d). One case (ID 141634) showed pseudoangiomatous spaces filled with blood and surrounded by tumor cells, a pattern that to some extend resembled an angiomatoid fibrous histiocytoma (Fig. 2e). Another case (ID 129604) showed prominent plasma cell aggregates and scattered eosinophils within an otherwise hypocellular tumor stroma, resembling inflammatory myofibroblastic tumor (Fig. 2f).

### Recurrent gene amplifications beyond *MDM2* and *PDGFRα*

**Fig. 3:**
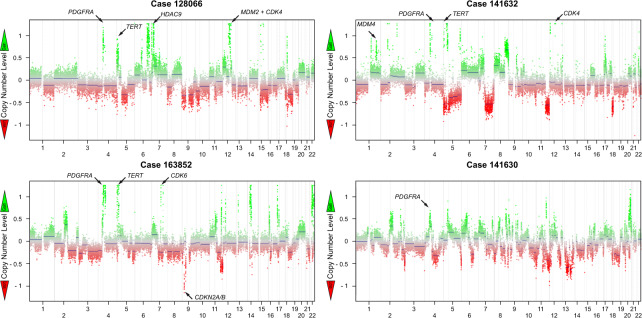
Copy number profiles in intimal sarcomas. Exemplary copy number profiles of intimal sarcomas carrying a 12q14-q15 amplification including *MDM2* and *CDK4* (upper left), carrying a 1q32.1 amplification including *MDM4* (upper right), carrying a 7q21.2 amplification including *CDK6* (lower left) and one example case lacking one of these gene amplifications (lower right).

**Fig. 4:**
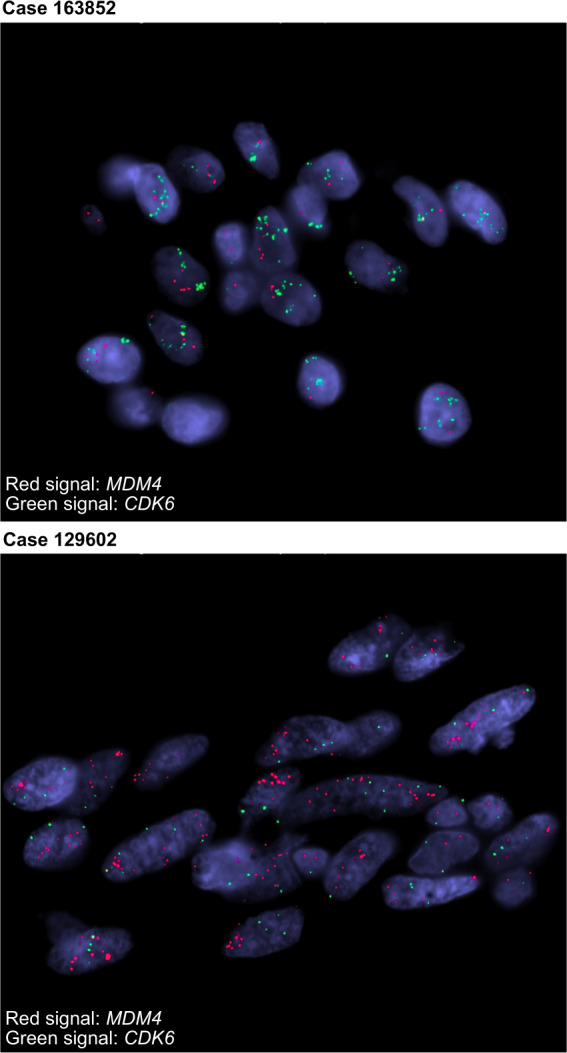
*MDM4* and *CDK6* amplifications demonstrated by FISH. 2-color FISH for *CDK6* (green signal) or *MDM4* (red signal) in *MDM2* balanced intimal sarcomas. The upper image (case 163852) shows tight clustered *green* signals (*CDK6*) and almost balanced red signals (*MDM4*). The lower image (case 129602) shows the opposite constellation with abundant red signals (*MDM4*) and almost balanced green signals (*CDK6*).

**Fig. 5:**
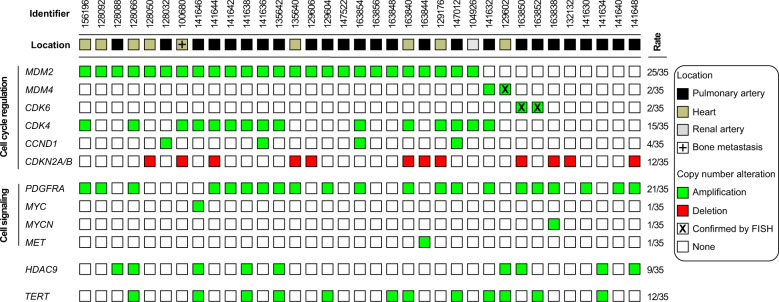
Summary of copy number variations in intimal sarcomas. Shown are the the most prominent copy number alterations identified. The color code is indicated in the figure.

We next analyzed the copy number profiles for amplifications and deletions (Fig. 3). ISAs and UPS of the left atrium showed complex karyotypes with overlapping patterns. Copy number analysis unveiled mutually exclusive amplifications in the cell cycle regulating genes *MDM2* (25/35), *MDM4* (2/35), and *CDK6* (2/35). *MDM4* and *CDK6* amplifications, where possible, were confirmed by FISH analysis (Fig. 4). Copy number variations in these pathways included co-amplifications of *CDK4* (15/35) and *CCND1* (4/35) and recurrent homozygous deletions in *CDKN2A/B* (10/35). Amplifications in signaling pathways most frequently involved receptor tyrosine kinase (RTK) *PDGFRA (21/35)* followed by *MET*, *MYC,* and *MYCN* (each 1/35). Furthermore, we observed recurrent amplifications in *TERT* (12/35) and *HDAC9* (9/35). The copy number variations are summarized in Fig. 5.

### Common DNA methylation signature in intimal sarcomas and undifferentiated pleomorphic sarcomas of the left atrium

**Fig. 6:**
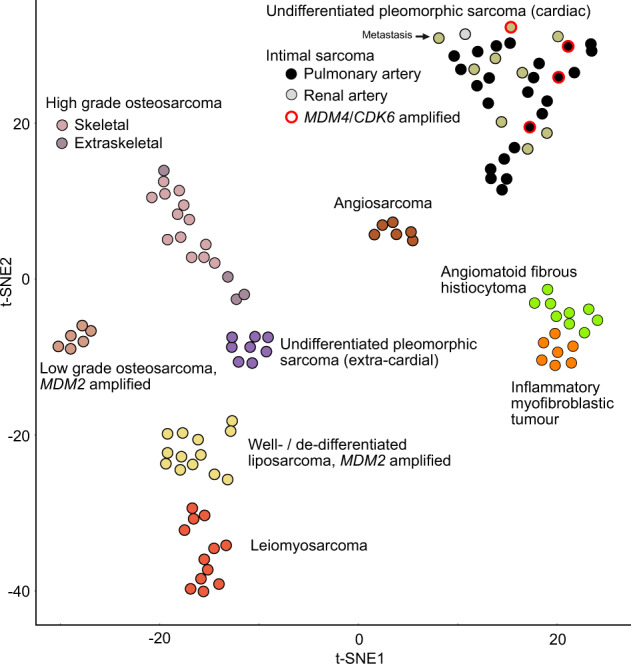
Unsupervised DNA methylation analysis of intimal sarcomas, undifferentiated pleomorphic sarcomas of the left atrium and potential mimics. Unsupervised t-Distributed Stochastic Neighbor Embedding (t-SNE) analysis of DNA methylation data from intimal sarcomas. The study cohort was compared with methylation data of prototypical soft tissue sarcomas that may histologically or molecularly mimic intimal sarcomas.

We next analyzed DNA methylation profiles by t-SNE analysis (Fig. 6). ISAs and UPS of the left atrium formed a unique methylation cluster when compared to potential histopathologic and molecular mimics, thereby demonstrating a distinct “ISA” methylation signature. Interestingly, the sample of a bone metastasis of an ISA overlaid with primary ISA samples, which illustrates the stability of this “ISA” methylation signature. Finally, it is important to note that the aforementioned mimics also constituted subtype specific clusters in the t-SNE plot.

## Discussion

In this study we assessed the molecular profiles of ISAs and UPS of the left atrium using genome-wide copy number profiling and unsupervised DNA methylation analysis. ISAs and UPS of the left atrium showed highly complex karyotypes. Our analysis revealed *MDM4* and *CDK6* amplifications in ISAs and cardiac UPS lacking *MDM2* amplifications. Furthermore, their epigenetic patterns were highly overlapping. Thus, our data argue in favor for the proposed concept by Neuville and colleagues that ISAs and UPS of the left atrium may constitute a common entity [13, 16].

Previous studies on ISAs and cardiac sarcomas have used array-CGH analysis only in a small number of samples and tested their findings on larger cohorts by fluorescence in situ hybridization analysis. These studies revealed complex karyotypes with highly recurrent amplifications of *MDM2* [8–15]. With this approach, however, there is a risk that less frequent copy number variations can be missed. In our study, all 35 cases were tested for genome-wide copy number variations. We could confirm *MDM2* amplifications in the vast majority of ISAs and additionally identified previously unreported, mutually exclusive *MDM4* and *CDK6* amplifications in ISAs and UPS of the left atrium. MDM4 and CDK6 immunohistochemistry were strongly positive in cases with such amplifications (data not shown). However, we also noticed a marked MDM4 and CDK6 expression in cases with balanced *MDM4* and *CDK6* locus. Therefore, we consider MDM4/CDK6 immunohistochemistry not suitable as surrogate for detecting MDM4/CDK6 amplifications.

MDM2 and CDK6 are critical components in the p53 and RB signaling pathway, respectively. These pathways are frequently disrupted in sarcomas with complex karyotypes, probably because subunits of those pathways are also involved in maintaining chromosome integrity [32]. CDK6 and CDK4 form a complex that suppresses RB1, a key element in the RB pathway. Notably, the co-amplification of *CCND1* and the recurrent deletion of *CDKN2A/B* also contribute to the disruption of the RB pathway [33]. MDM4 is best characterized for repressing p53 transactivation activity and p53 translational regulation in normal cells under stress [34]. Because of the high frequency of amplifications in these cell cycle pathways, they appear to contribute to the pathogenesis in ISA and UPS of the left atrium.

We also observed highly recurrent amplifications of *PDGFRA*, as previously reported [12]. Other RTKs were only rarely amplified, e.g., *MET*. Copy number alterations were rarely observed in the oncogenes *MYC* and *MYCN*. Furthermore, we observed frequent *TERT* amplifications, which confer unlimited proliferation potential to cancer cells through telomere length maintenance. *TERT* amplifications have been shown to confer the highest telomerase activity among tumors [35]. Finally, we detected *HDAC9* amplifications in ISAs. Higher expression of *HDAC9* has been associated with poor prognosis in different cancer types [36]. These oncogene amplifications in ISAs and UPS of the left atrium may qualify patients for targeted therapies [37, 38]. However, it remains to be determined whether targeted therapies can lead to a treatment response in ISAs [2].

Some cases lacking *MDM2*, *MDM4,* or *CDK6* amplifications remain ambiguous by copy number profiling. It is conceivable that these cases harbor alternative driver mutations, although ISAs show a relative low mutation rate overall [15]. In our study, cases lacking *MDM2*, *MDM4,* or *CDK6* amplifications often carried *PDGFRA* amplifications. However, *PDGFRA* amplifications are among the most common genetic alterations in cancer. Accordingly, we observed *PDGFRA* amplifications also in 5/12 leiomyosarcomas (data not shown). Hence, the detection of *PDGFRA* amplifications alone is non-specific for diagnosing ISA [39]. To some extent, the same concerns apply for *MDM2/4* amplifications. ISAs of uncommon sites such as the retroperitoneum, where dedifferentiated liposarcoma is always a strong consideration, are prone to be misdiagnosed, especially in conjunction with an underlying *MDM2* amplification [40–42]. Thus, clinical correlation is paramount for the diagnosis of ISAs and the isolated use of established molecular markers alone may not be helpful in this context.

We provide evidence that DNA methylation profiling may be valuable in increasing the diagnostic accuracy of these rare tumors. Our methylation analysis led to the identification of a common methylation fingerprint in ISAs and cardiac UPS. We assume this “ISA” methylation signature to be specific. Accordingly, relevant differential diagnoses, e.g., dedifferentiated liposarcomas and extra-cardiac UPS showed distinct methylation signatures compared to ISA [43, 44]. Notably, these methylation signatures remain stable over the timeline of the disease, as previously reported in other tumor entities [45]. We could detect the ISA methylation signature even in the bone metastasis of a cardiac UPS. In view of the specificity and stability of this “ISA” DNA methylation signature, DNA methylation profiling may be a valuable ancillary biomarker in ISAs and UPS of the left atrium, especially in cases lacking *MDM2*, *MDM4* or *CDK6* amplifications or occurring at unusual sites.

## Supplementary information

Supplementary Table 1

## Data Availability

The data are available from the corresponding author upon request.
